# Validation study of small-angle X-ray scattering tensor tomography

**DOI:** 10.1107/S1600577520003860

**Published:** 2020-04-22

**Authors:** Manuel Guizar-Sicairos, Marios Georgiadis, Marianne Liebi

**Affiliations:** a Paul Scherrer Institute (PSI), 5232 Villigen, Switzerland; b Institute for Biomechanics, ETH Zurich, 8093 Zurich, Switzerland; c Stanford Medicine, Stanford University, Stanford, CA 94305, USA; d Department of Physics, Chalmers University of Technology, 41296 Gothenburg, Sweden

**Keywords:** SAXS, tensor tomography, nanostructure orientations

## Abstract

Small-angle scattering tensor tomography (SASTT) is a recently developed technique able to tomographically reconstruct the 3D reciprocal space from voxels within a bulk volume. SASTT extends the concept of X-ray computed tomography, which typically reconstructs scalar values, by reconstructing a tensor per voxel, which represents the local nanostructure 3D organization. In this study, the nanostructure orientation in a human trabecular-bone sample obtained by SASTT was validated by sectioning the sample and using 3D scanning small-angle X-ray scattering (3D sSAXS) to measure and analyze the orientation from single voxels within each thin section. Besides the presence of cutting artefacts from the slicing process, the nanostructure orientations obtained with the two independent methods were in good agreement, as quantified with the absolute value of the dot product calculated between the nanostructure main orientations obtained in each voxel. The average dot product per voxel over the full sample containing over 10 000 voxels was 0.84, and in six slices, in which fewer cutting artefacts were observed, the dot product increased to 0.91. In addition, SAXS tensor tomography not only yields orientation information but can also reconstruct the full 3D reciprocal-space map. It is shown that the measured anisotropic scattering for individual voxels was reproduced from the SASTT reconstruction in each voxel of the 3D sample. The scattering curves along different 3D directions are validated with data from single voxels, demonstrating SASTT’s potential for a separate analysis of nanostructure orientation and structural information from the angle-dependent intensity distribution.

## Abstract 2

Small-angle scattering tensor tomography allows for tomographic reconstruction of the 3D reciprocal space from voxels within a bulk volume. In this study, the nanostructure orientation and the scattering curve along different 3D directions are validated with data obtained from thin sections of the same sample.

## 1. Introduction   

Scattering techniques are powerful tools for studying the orientation of anisotropic building blocks (Fratzl *et al.*, 1993 ▸; Guinier & Fournet, 1955 ▸; Georgiadis *et al.*, 2016*a* ▸). Small-angle X-ray scattering (SAXS) probes the spatial variation of electron density at the nanometre scale, thus anisotropy of the measured scattering pattern can be related to the orientation of the sample’s nanostructure. Some examples from materials science and biology are the alignment of cellulose fibrils in wood (Lichtenegger *et al.*, 1999 ▸; Fratzl *et al.*, 1997 ▸), oriented structures in semi-crystalline polymers (Schrauwen *et al.*, 2004 ▸; Stribeck *et al.*, 2008 ▸; Tang *et al.*, 2007 ▸), alignment of carbon nanotubes in films (Wang *et al.*, 2007 ▸) or the arrangement of mineralized collagen fibrils in bone (Pabisch *et al.*, 2013 ▸; Fratzl *et al.*, 1996 ▸). A single measurement on a 2D detector is inherently limited to capturing the 2D information of the underlying 3D orientation. To obtain the full 3D orientation distribution, repeated measurements while rotating the sample with respect to the X-ray beam are needed (Seidel *et al.*, 2012 ▸; Liu *et al.*, 2010 ▸; Georgiadis *et al.*, 2015 ▸). For non-homogeneous samples, scanning SAXS (sSAXS) with a small beam can capture the nanostructure arrangement distribution over the samples’ volume (Pabisch *et al.*, 2013 ▸; Fratzl *et al.*, 1997 ▸; Bunk *et al.*, 2009 ▸; Paris, 2008 ▸). For isotropically scattering samples, sSAXS can be directly combined with computed tomography (CT) using standard reconstruction techniques such as filtered back projection or algebraic reconstruction techniques in each scattering angle, *i.e.* momentum transfer *q*, individually (Jensen *et al.*, 2011*a* ▸,*b* ▸; Álvarez-Murga *et al.*, 2012 ▸); a similar approach for the wide-angle scattering regime is used in diffraction tomography (Birkbak *et al.*, 2015 ▸). The standard tomography reconstruction methods can also be used for anisotropic samples as long as there is structural symmetry around the rotation axis, rendering the scattering invariant with respect to sample rotation (Stribeck *et al.*, 2006 ▸; Feldkamp *et al.*, 2009 ▸; Schroer *et al.*, 2006 ▸).

Recent developments have enabled the tomographic reconstruction of the orientation for anisotropically scattering samples (Skjønsfjell *et al.*, 2016 ▸; Schaff *et al.*, 2015 ▸; Liebi *et al.*, 2015 ▸, 2018 ▸; Gao *et al.*, 2019 ▸). Under strict assumptions on the sample, such as known dimensions of the scattering particles and slowly varying orientation confined in one plane, the orientation distribution can be obtained from a single rotation axis (Skjønsfjell *et al.*, 2016 ▸). For general anisotropically oriented scatterers, two rotation axes are used to retrieve the 3D reciprocal space (Schaff *et al.*, 2015 ▸; Liebi *et al.*, 2015 ▸). Three numerical approaches have been demonstrated for reconstruction of the full 3D reciprocal-space map. The first method consists of multiple independent CT reconstructions of the scattering contribution parallel to so-called virtual tomography axes (Schaff *et al.*, 2015 ▸). This method is based on an extension of the concept of rotation invariance (Feldkamp *et al.*, 2009 ▸), where SAXS patterns are acquired at different sample rotations around two axes. In processing, the data are grouped into subsets that correspond to scattering parallel to a virtual tomography axis, within a defined error threshold for the sample orientation angles. Scattering intensity along these virtual axes directions is retrieved for each voxel. The full 3D reciprocal-space map can also be reconstructed using a series of spherical harmonics as a model representing the 3D scattering distribution in each voxel (Liebi *et al.*, 2015 ▸), a method we will refer to as small-angle scattering tensor tomography (SASTT) in the following. Under the assumption of having a single preferential nanostructure orientation per *q* range and voxel, as well as cylindrical symmetry of the nanostructure, the number of spherical harmonic functions needed to represent the 3D reciprocal space can be drastically reduced by optimizing over the orientation of the spherical harmonics zenith direction, which is parameterized by two spherical angles (Liebi *et al.*, 2015 ▸, 2018 ▸). Finally, a recently introduced reconstruction technique termed iterative reconstruction tensor tomography (IRTT) uses a second-rank tensor model for describing the orientation distribution function in each voxel (Gao *et al.*, 2019 ▸). While the second-rank tensor model introduces some limitations in the complexity of features that can be represented, IRTT has been shown to be fast and robust. A comparison and cross-validation between IRTT and SASTT is given in the work of Gao *et al.* (2019 ▸).

**Figure 1:**
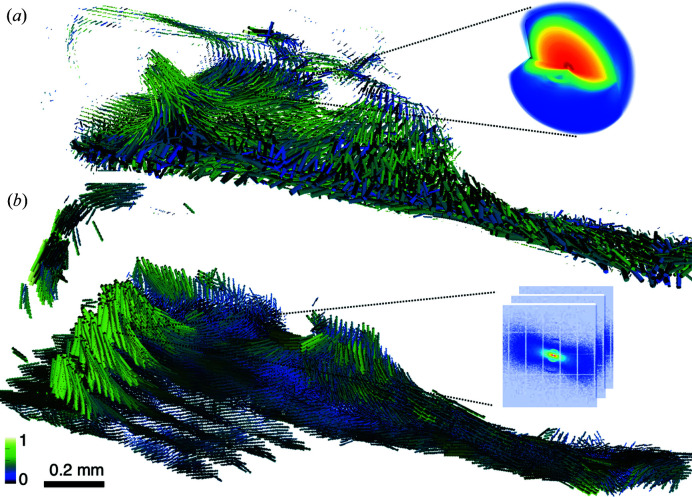
(*a*) In SAXS tensor tomography (Liebi *et al.*, 2015 ▸) the full 3D reciprocal-space map in each voxel can be determined, from which we can retrieve the 3D nanostructure preferential orientation and degree of orientation, represented by cylinder orientation and colour, respectively. (*b*) The reconstruction was validated by sectioning the sample and measuring each slice under different sample rotations; the resulting series of 2D SAXS patterns for each voxel is used to determine the 3D orientation and degree of orientation via 3D sSAXS (Georgiadis *et al.*, 2015 ▸). The colour bar shows the degree of orientation normalized with the maximal degree of orientation calculated by each method.

In this article, we validate the SASTT reconstruction by comparing the orientations of mineralized collagen fibrils from the human trabecular-bone sample presented earlier (Liebi *et al.*, 2015 ▸), shown in Fig. 1 ▸(*a*), with the orientations obtained after slicing the same sample and re-measuring and analyzing each thin section as described in the work of Georgiadis *et al.* (2015 ▸) [Fig. 1 ▸(*b*)] and referred to as 3D sSAXS in the following. By measuring thin sections of the sample, slices of each voxel’s 3D reciprocal-space map are directly measurable in the scattering patterns. In addition, we compare directly the reconstructed 3D reciprocal-space map from a voxel within the intact 3D sample with the measured anisotropic scattering from that single voxel after slicing the sample. This approach is akin to validating CT with histology slices, but here it is carried out with tensors instead of scalar values because of the nature of the recovered information. In addition, we validate the regularization on the orientation direction, which is used in the SASTT reconstruction to suppress high-spatial-frequency noise (Liebi *et al.*, 2018 ▸), by comparing results with and without regularization, showing that the use of regularization provides a better correlation with the orientations obtained using 3D sSAXS.

## 2. Validation of nanostructure orientation   

**Figure 2:**
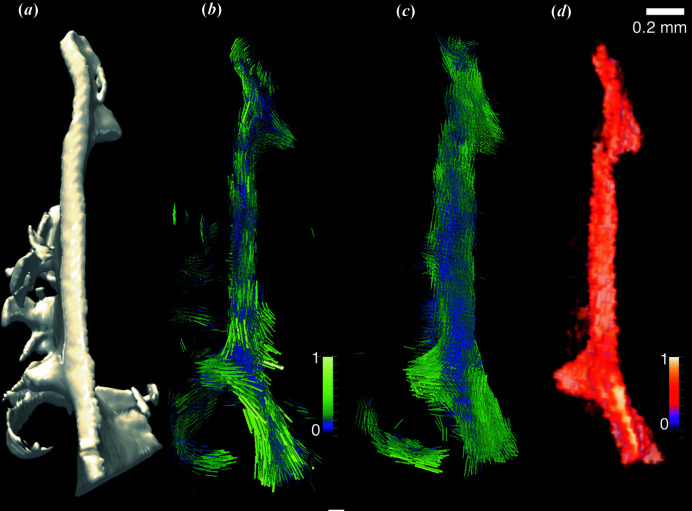
(*a*) Absorption-based volume rendering from µCT of the human trabecular-bone intact 3D sample, performed after SASTT scanning. (*b*) SAXS tensor tomography on the sample showing the orientation and normalized degree of orientation, the latter length and colour coded, of mineralized collagen fibres. (*c*) 3D sSAXS of the same trabecula from 38 consecutive sections stacked up. (*d*) Absolute value of the dot product between the orientation obtained from the SAXS tensor tomography and 3D sSAXS of the sectioned sample; dot product = 1 for perfect agreement between the two methods.

The sample is the trabecula of a human vertebra of 1 mm × 1 mm × 2.5 mm measured in the work of Liebi *et al.* (2015 ▸). Fig. 2 ▸(*a*) shows a volume rendering based on absorbance information from conventional micro-computed tomography (µCT). Reconstruction from SASTT (Liebi *et al.*, 2015 ▸), shown in Fig. 2 ▸(*b*), reveals the bone ultrastructure in each voxel of 25 µm × 25 µm × 25 µm. The same sample was cut into sections with 20 µm thickness and 38 consecutive sections were measured using 3D sSAXS (Georgiadis *et al.*, 2015 ▸), revealing the organization of their 3D ultrastructure. The sections were aligned and registered to the 3D sample, as shown in Fig. 2 ▸(*c*), in order to validate the ultrastructure orientation obtained from SASTT; for more details see Appendix *A* [App appa]. The orientation vector in each voxel is represented by the orientations of the cylinders rendered in Figs. 2 ▸(*b*) and 2 ▸(*c*), whereas the degree of orientation is represented by the colour bar.

**Figure 3:**
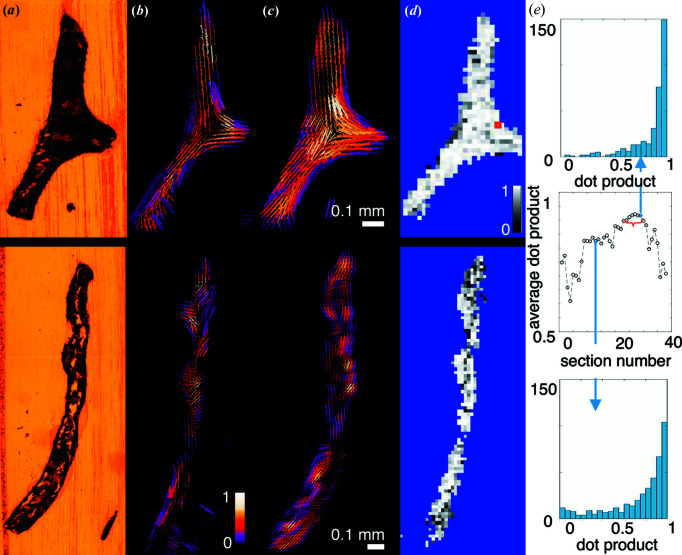
Comparison of results from two selected slices. (*a*) Microscopy image showing that the sample has been damaged by slicing to a small (top) or high (bottom) degree. Reconstruction results from (*b*) SASTT and (*c*) 3D sSAXS on the same virtual and physical slice, respectively. The cylinders represent the direction of the main orientation; the length represents the degree of orientation and the colour represents the symmetric intensity, normalized with the maximum value. (*d*) Map of the dot product calculated between the vectors from the two methods and (*e*) the corresponding histogram of the dot product as well as the average dot product as a function of the section number, marking the position of the two selected sections shown here, and the region of sections where fewer cracks occurred (red bracket). The red square in (*d*) marks the voxel where the *q*-resolved reconstruction, shown in Fig. 5 ▸, was validated.

In order to quantify the agreement of the nanostructure orientations of both methods, we registered the volumes and computed a dot product between the retrieved orientation unit vectors for each voxel; a dot product of 1 represents perfect agreement between the orientations determined with the two methods. A colour-coded volume rendering of the dot product is shown in Fig. 2 ▸(*d*). Comparing the overall shape of the sample, there is good agreement between µCT (*a*) and SASTT (*b*), whereas the sample volume obtained from 3D sSAXS appears larger. This indicates the presence of cutting artefacts for 3D sSAXS which results in problems with the registration of the sections to the 3D sample. However, high dot-product values suggest there is a good overall agreement between the nanostructure orientations obtained with the two methods. In order to better compare the orientations obtained in each voxel, two selected slices are shown in Fig. 3 ▸.

The ultrastructure orientation determined from 3D sSAXS of two sections is shown in Fig. 3 ▸(*b*) and the ultrastructure orientation reconstructed from SAXS tensor tomography is shown in the corresponding virtual slices in Fig. 3 ▸(*c*). Like in Fig. 2 ▸, the cylinders represent the direction of the main scattering and the cylinder length represents the degree of orientation, whereas the colour represents the symmetric scattering intensity. The sections clearly show cracks visible in the microscopic images, shown in Fig. 3 ▸(*a*), which are more pronounced in the section displayed in the bottom row. Since the cracks did not appear in the SAXS tensor tomography [Fig. 2 ▸(*b*)] or in the µCT [Fig. 2 ▸(*a*)], they are most likely induced by the slicing process with the microtome. The appearance of cracks together with related registration issues also explains the apparently larger 3D volume composed from the 38 sections [Fig. 2 ▸(*c*)] compared with the volume measured from the intact 3D sample [Figs. 2 ▸(*a*) and 2(*b*)].

It is possible that cracking occurred more frequently during slicing because of an increased brittleness of the sample induced by radiation in the SASTT and subsequent µCT measurements, since mechanical properties are reported to change already at low dosage (Barth *et al.*, 2010 ▸, 2011 ▸), which with an estimated dose of 2.9 × 10^7^ Gy (Liebi *et al.*, 2015 ▸) has been exceeded in the SASTT measurement. Furthermore, the slicing of a single trabecula in a poly-methyl-methacrylate (PMMA) block is even more challenging than a larger bone volume embedded in PMMA because of the mismatch of hardness between bone and the PMMA matrix.

However, even though the sample is partly damaged, the directions of mineralized ultrastructure agree well in the regions that are intact, which can be qualitatively seen comparing Fig. 2 ▸(*b*) with Fig. 2 ▸(*c*) and Fig. 3 ▸(*b*) with Fig. 3 ▸(*c*). Quantitatively, this can be seen in the calculated dot product between the 3D vectors obtained by the two methods [Figs. 2 ▸(*d*) and 3 ▸(*d*), and the histograms in Fig. 3 ▸(*e*)]. The agreement of the obtained ultrastructure orientation is better, *i.e.* with higher dot product as shown in Fig. 3 ▸(*e*), in sections which show less cracks and macroscopic damage, as can be expected. For instance, the section in the top row of Fig. 3 ▸(*a*) shows less cracks, and the better agreement between the two methods can be seen visually in Figs. 3 ▸(*b*) and 3 ▸(*c*), and also by the histograms in Fig. 3 ▸(*e*). The average dot product per voxel over the full trabecula was 0.84, calculated from 12 059 voxels. In the sections where less cracks occurred, sections 24–29 marked with a red bracket in Fig. 3 ▸(*e*), the average dot product per voxel increased to 0.91, calculated from 2623 voxels, with 57% of the voxels showing less than 20° deviation between the 3D orientations obtained with the two methods (dot product > 0.94 = cos 20°). The calculation of the dot product did not include a weighing with the degree of orientation.

## 3. Validation of orientation regularization   

**Figure 4:**
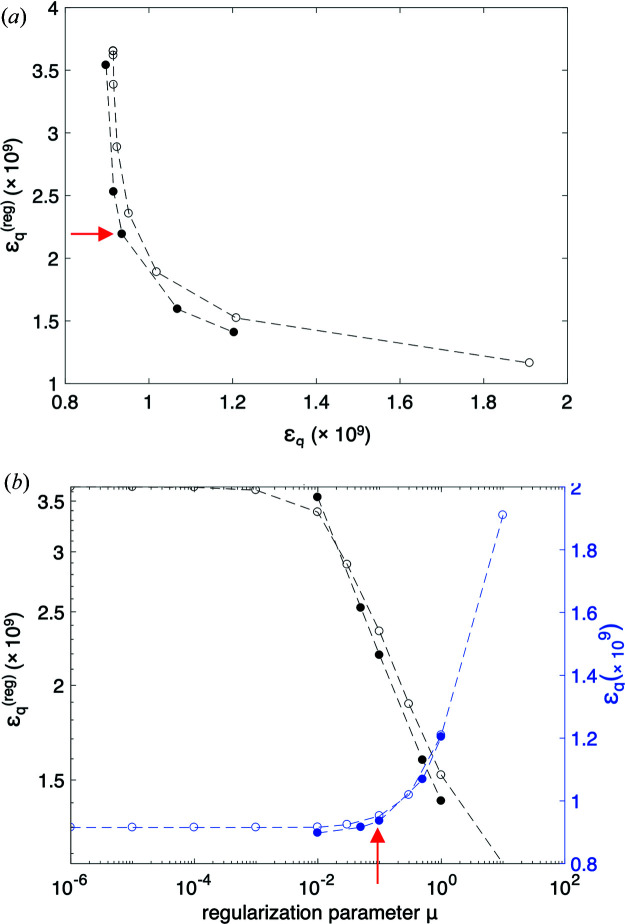
Effect of regularization of orientation in the SASTT optimization, as introduced in the work of Liebi *et al.* (2018 ▸). (*a*) The L-curve method is used to find the appropriate regularization parameter, in this case μ = 0.1 (marked with a red arrow). (*b*) Corresponding dependence of penalty term ∊_*q*_^(reg)^ (left vertical axis, black) and of the error metric ∊_*q*_ (right vertical axis, blue) on the regularization parameter μ. Open circles refer to optimization with five iterations, whereas the full circles refer to an optimization with 50 iterations.

In addition, we validated the orientation regularization applied in the optimization of the nanostructure orientation, as introduced in the work of Liebi *et al.* (2018 ▸). The regularization acts on the spherical harmonics azimuth orientation, which is parametrized in each voxel by the polar and azimuthal angles θ_op_ and φ_op_. We introduce an additional regularization term in the error metric ∊_*q*_^(reg)^, which is weighted by the regularization coefficient μ. We have previously shown that this is an effective measure to mitigate high-frequency noise on the 3D orientation (Liebi *et al.*, 2018 ▸). The optimal regularization coefficient μ is determined with the L-curve technique (Santos & Bassrei, 2007 ▸; Hansen, 1992 ▸; Belge *et al.*, 2002 ▸), in which we compare the regularization error metric term versus the data error metric for a range of values of μ. The corner of the L curve therefore corresponds to the regularization coefficient for which there is a notable gain in regularization without a significant increase of the data error metric, thereby favouring solutions with smoother orientation distributions. Fig. 4 ▸(*a*) shows the L curve for the trabecular-bone sample studied here, Fig. 4 ▸(*b*) shows the error metric ∊_*q*_ and the regularization term of the error metric ∊_*q*_^(reg)^ as a function of the regularization parameter μ. Open circles represent optimizations with only five iterations, which show the same dependence of ∊_*q*_ and ∊_*q*_^(reg)^ on μ than the full circles corresponding to optimizations with 50 iterations. This indicates that the optimal regularization coefficient can be robustly determined even with a small number of iterations, reducing computing time significantly. The red arrow marks the regularization coefficient μ = 0.1, which introduces an effective regularization without significant increase in the error metric ∊_*q*_. This value is chosen at the left of the L corner to favour lower error metric versus smoothing.

**Figure 5:**
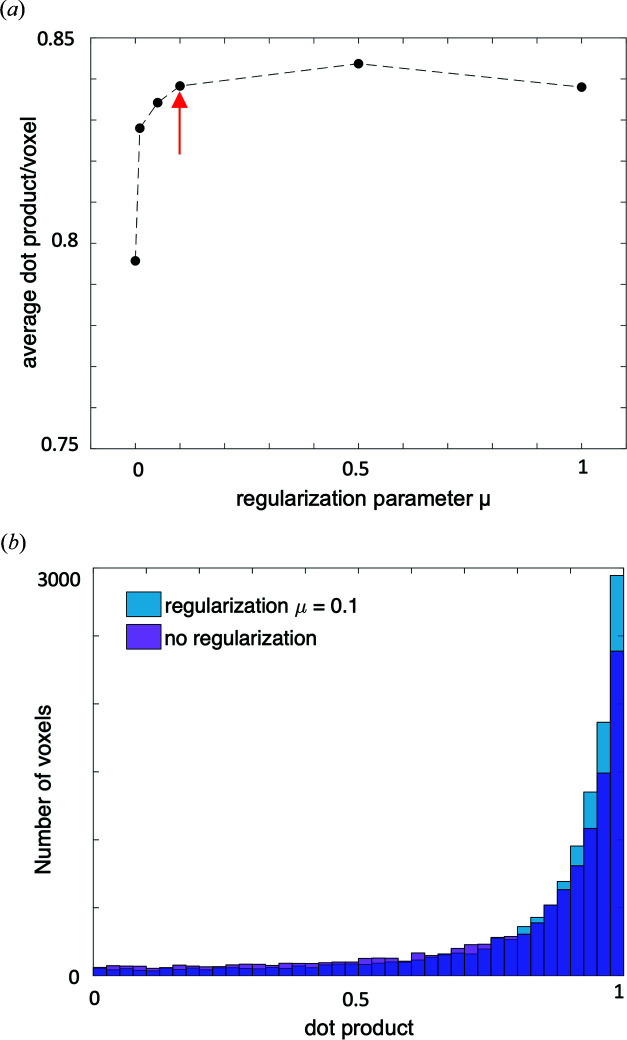
(*a*) Average dot product versus regularization coefficient μ. The red arrow marks μ = 0.1, identified by the L curve as the appropriate regularization coefficient. The dot product is calculated between nanostructure orientations retrieved from SASTT on the intact sample and those obtained using 3D sSAXS on the sliced sample. (*b*) Corresponding histogram for μ = 0 and μ = 0.1.

Fig. 5 ▸(*a*) shows the average dot product of the orientation obtained by 3D sSAXS on the sectioned sample with the orientation from an optimization with varying regularization coefficient μ. The average dot product per voxel increases from 0.79, without regularization, to a fairly constant value of 0.84 for μ > 0.1. The improved agreement using regularization is supported by the histogram of all dot products in the sample, shown in Fig. 5 ▸(*b*), which is shifted towards larger values with regularization. We confirm therefrom that the L-curve method, as was introduced in the work of Liebi *et al.* (2018 ▸), is an appropriate method for determining the regularization coefficient μ. Selecting a point at the left-side corner of the L curve [red arrow in Fig. 4 ▸(*a*)] prioritizes a smaller error over a smooth solution.

## 4. Validation of *q*-resolved reconstruction   

One of the main advantages of SASTT is its ability to probe properties of anisotropic nanostructure locally, in 3D, and within bulk samples. To show this, we compare a *q*-resolved reconstruction in one voxel with SAXS data acquired on the corresponding slice, at two different scattering orientations. In order to do this, we identify the voxel corresponding to the section data, and from the reciprocal-space map reconstruction we extract the intensity at an orientation that matches the 3D sSAXS experiments on sections; in this way, we can use the SASTT reconstruction to predict the scattering of the section at this orientation and are able to compare directly our reconstruction of the reciprocal-space map with the data measured on the sectioned sample.

**Figure 6:**
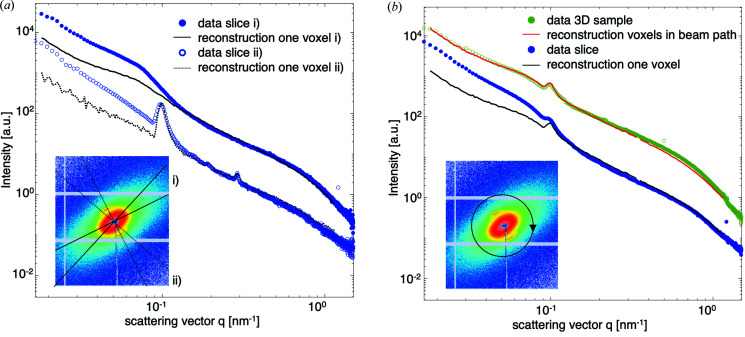
Measured versus reconstructed *q*-resolved anisotropic small-angle scattering. (*a*) Curves from a selected point of a slice [marked red in Fig. 3 ▸(*d*)] in two different azimuthal angles, i) and ii), as defined by the black lines in the inset diffraction pattern, measured with the section oriented perpendicular to the X-ray beam. The lower curves depict intensities in the direction that includes the collagen peak, segment i), with blue open circles representing the data from the inset diffraction pattern, *i.e.* the section, and the dotted black line represents the reconstructed data for this voxel in the corresponding direction. The upper curves depict *q*-resolved scattering along the direction perpendicular to the collagen peak, segment ii), which has higher scattering for all *q* values, a blue curve for the section and a black curve for reconstructed data. (*b*) The lower curves correspond to the azimuthally integrated *q*-resolved data from the same diffraction pattern as in (*a*), with the section data in blue and corresponding *q*-resolved reconstruction in the corresponding voxel in black. The green circles show azimuthally integrated (measurement) data from the SASTT scan through the same point, thus including scattering from all voxels in the beam path. The red curve shows the *q*-resolved corresponding reconstruction, where contributions from all voxels along the same beam path have been summed.

Fig. 6 ▸(*a*) shows the comparison between a SAXS pattern from the sliced sample measured with the section perpendicular to the X-ray beam, shown with blue circles, and the calculated intensity from the reconstructed reciprocal-space map of the corresponding voxel, shown by black lines; the voxel is marked by a red square in Fig. 3 ▸(*d*). The comparison is shown for two different azimuthal directions on the detector, which are indicated in the inset diffraction pattern. Scattering at high *q* values (*q* > 0.2 nm^−1^), related to the thickness of mineral platelets, could be reproduced with good agreement. The peak from the collagen *d* period, produced when its orientation fulfils the Bragg condition, was also reproduced.

At low *q* values, there is an increased scattering intensity in the measured data from slices, which is not present in the reconstructed data. This can also be observed in the comparison of the azimuthally integrated intensities shown in Fig. 6 ▸(*b*), with blue circles for the data and with a black line for the SASTT-calculated intensity; these curves practically overlap at high *q* values and differ at low *q* values, with the section data having higher intensity. To ensure that this lower SASTT-retrieved intensity does not result from improper convergence of the SASTT reconstruction, we show in Fig. 6 ▸(*b*) a comparison of the azimuthally integrated raw (measured) data from SASTT, in green open circles, versus the (calculated) azimuthally integrated reconstruction data, summed along all voxels of the same beam path, shown by a red line; the reconstruction appears to be in good agreement with the raw SASTT data and does not show a deficit of intensity. This indicates that, rather than a problem of reconstruction, an increased scattering at low *q* values is measured for the sectioned sample as compared with the intact 3D sample. Possible reasons for the difference could be dust or other structured contamination present on the slices which would lead to an increased forward scattering, likewise would the presence of small cracks or modifications of the nanostructure induced by cutting, which would scatter with *I* ∝ *q* ^−4^ and thus contribute more at low *q* values. In addition, the Kapton tape on which the slices were mounted adds to the increased scattering at low *q* values; however, this background scattering is two order of magnitudes lower and thus contributes only marginally.

## 5. Conclusions   

In the first demonstration of SAXS tensor tomography (Liebi *et al.*, 2015 ▸), the orientation of mineralized collagen fibrils in a bone trabecula was obtained. The result therein was judged to be reasonable as the observed ultrastructure was in agreement with general rules of collagen orientation in trabecular-bone samples (Georgiadis *et al.*, 2016*b* ▸). Here we presented a validation study of SAXS tensor tomography by comparing it with a method for which the trabecula was cut into thin sections and each of them measured under different rotations relative to the X-ray beam. The 3D orientation of ultrastructure within each of these sections was obtained using 3D sSAXS (Georgiadis *et al.*, 2015 ▸). The two independent determinations of ultrastructure orientation were compared by calculating the dot product, see Fig. 3 ▸, resulting in an average dot product per voxel in the region with less cutting artefacts of 0.91, with 57% of the voxels showing less than 20° deviation between the 3D orientations obtained with the two methods. As expected, when the obtained 3D orientations were compared visually, both cases showed that the ultrastructure orientation follows the curvature of the trabecular bone (Georgiadis *et al.*, 2016*b* ▸). A clear limitation of the validation study presented here was that the sample suffered from cracking in the sectioning procedure, which caused difficulties in the registration of the sections to the 3D sample. However, the two methods still agree well in the obtained ultrastructure orientation.

This study also highlights the advantage of SAXS tensor tomography compared with measurements performed on thin slices, by eliminating the need for destructive sectioning, thus reducing the risk of slicing artefacts or making measurements feasible in the first place for samples which are too fragile or valuable to be sliced.

The regularization in the optimization, which was introduced to reduce high-frequency noise in the reconstruction of the nanostructure orientation (Liebi *et al.*, 2018 ▸), is shown here to increase the agreement with the directions obtained by 3D sSAXS on the sliced sample; see Fig. 6 ▸.

SASTT not only provides the 3D nanostructure orientation but also the full 3D reciprocal-space map. In particular, it allows one to obtain the *q*-resolved anisotropic scattering. Thus, in each voxel of the 3D sample, the entire scattering curve in any possible illumination direction can be determined. Here, we have obtained the anisotropic scattering of one voxel and compared it with the measured scattering from the same voxel scanned at the corresponding section. There was good agreement of the single-voxel measurement and SASTT-reconstructed scattering, apart from deviations in the low-*q* region. The disagreement is attributed to be an artefact from measuring slices that include small cracks and being mounted on Kapton tapes, whereas the reconstruction from SASTT is based on measurements on the intact 3D sample. The obtained intensity versus *q* curves can be analyzed using standard analysis tools, such as peak fitting, or fitting models to retrieve structural parameters such as the thickness of mineral platelets (Fratzl *et al.*, 2005 ▸). The possibility to reproduce the anisotropic scattering in any direction opens up new routes of analyzing SAXS data. In SASTT, as presented in the work of Liebi *et al.* (2015 ▸), the local preferential orientation is parameterized with a unit vector, thus it is straightforward to decouple the orientation effect from the *q*-dependent scattering intensity profile. Further analysis of the intensity profile can thus be performed either on different 3D directions, *e.g.* parallel or perpendicular to the main scattering orientation, or the intensity can be averaged over any circle or even the full sphere in each *q* range. This is in contrast to standard SAXS measurements on anisotropic systems, where the measured scattering highly depends on the orientation of the sample in the beam, since the 2D SAXS pattern only reveals 2D information depending on the underlying 3D nanostructure orientation.

While this validation is limited to scattering models with cylindrical symmetry and a single preferential orientation per voxel, the spherical harmonics basis can represent much more general distributions if higher orders are included, as has been demonstrated for texture analysis (Roe, 1965 ▸, Bunge & Roberts, 1969 ▸) and more recently for directional dark-field tomography in the work of Wieczorek *et al.* (2016 ▸).

The present validation study aims to further demonstrate the potential of SASTT to be used in quantitative analyses, by isolating the scattering of local nanostructure with minimal invasive preparation. We believe that combined with developments in SASTT-related equipment such as faster encoder-equipped motors, increased data readout and transfer speeds, and higher fluxes in new generation synchrotrons, as well as with improved sampling schemes, the measurement time can be drastically reduced, which will increase the accessibility of the method.

## Appendix A Materials and methods

### A1. Sample preparation

A piece of trabecular bone was extracted using a scalpel from the 12th thoracic (T12) human vertebra of a 73-year-old man, cleaned from soft tissue and embedded into PMMA (Liebi *et al.*, 2015 ▸). The vertebra was obtained from the Department of Anatomy, Histology and Embryology at the Innsbruck Medical University, Innsbruck, Austria, with the written consent of the donor according to Austrian law. All procedures following were performed in accordance with Swiss law, the Guideline on Bio-Banking of the Swiss Academy of Medical Science (2006) and the Swiss ordinance 814.912 (2012) on the contained use of organisms. After the SAXS tensor tomography measurement, which has been presented in the work of Liebi *et al.* (2015 ▸), the sample was imaged with µCT. Subsequently, the sample was re-embedded in PMMA and cut into 20 µm-thin consecutive sections using a microtome (HM 355S; Thermo Fisher Scientific Inc., USA). During slicing, consecutive sections were directly mounted on 12 µm-thick Kapton adhesive tape (Benetec, Switzerland).

### A2. Experiments

Micro-CT was carried out in the Institute for Biomechanics of ETH Zurich, with 12.1 µm voxel size (µCT 40; Scanco Medical, Brüttisellen, Switzerland).

All X-ray scattering measurements were performed at the cSAXS beamline (X12SA) at the Swiss Light Source, Paul Scherrer Institute, Switzerland. A fixed-exit double-crystal Si(111) monochromator was used to yield a 12.4 keV X-ray beam. The beam was focused horizontally by bending the second monochromator crystal and focused vertically by bending a Rh-coated mirror. A 7 m-long flight tube under vacuum was placed between the sample and the Pilatus 2M detector (Kraft *et al.*, 2009 ▸), and the direct beam was recorded with a photodiode mounted on the beam stop inside the flight-tube. SAXS tensor tomography was measured using the setup and acquisition scheme described in the work of Liebi *et al.* (2015 ▸). 3D sSAXS on the thin sections was measured as described by Georgiadis *et al.* (2015 ▸). The scanning of each section was repeated at nine different rotation angles β (−60°, −30°, 0°, 30°, 60°, 135°, 165°, 195°, 225°) with an exposure time of 30 ms per SAXS pattern.

The beam size was 25 µm × 25 µm in SASTT and 20 µm × 20 µm in 3D sSAXS, the latter in order to match the section thickness and provide an isotropic voxel size for 3D sSAXS (Georgiadis *et al.*, 2015 ▸). The *x* and *y* motor step size was 25 µm × 25 µm for SAXS tensor tomography, and 20 × cos(β) µm × 20 µm for 3D sSAXS, where the adjustment of the step size in *x* by cos(β) for 3D sSAXS was chosen in order to have a consistent number of scanning points across the rotating specimen for all sample rotation angles.

### A3. Data analysis

The SASTT reconstruction was performed following the procedure described in the work of Liebi *et al.* (2015 ▸) and in more detail in the work of Liebi *et al.* (2018 ▸). The 3D reciprocal-space map in each voxel and in each *q*-range was thereby modelled with a series of spherical harmonics parameterized by the spherical harmonics coefficient *a*_l_^m^ and the local preferential orientations θ_op_ and φ_op_, and the reconstruction was carried out in a step-wise approach to avoid local minima (Liebi *et al.*, 2018 ▸). For the derivation of the nano­structure orientation, the reconstruction was performed on data integrated over a *q* range of 0.0379–0.0758 nm^−1^. In the same *q* range, 3D sSAXS, as described in the work of Georgiadis *et al.* (2015 ▸), was used to recover the 3D nanostructure orientation. For reconstruction of the complete trabecula from the consecutive sections, the transmission images from the scan at rotation β = 0° were registered to that of the preceding section using a sub-pixel image registration algorithm (Guizar-Sicairos *et al.*, 2008 ▸). The reconstructed 3D volumes from SAXS tensor tomography and from 3D sSAXS were rigidly registered to the 3D tomogram from µCT, from which the rotations around *x*, *y* and *z* and translations between the datasets were obtained. The 3D sSAXS volume was used as a reference coordinate system. The unit vector of the orientation **û** ^str^, as described in the work of Liebi *et al.* (2018 ▸), is used in the following steps to represent the nanostructure principal orientation. The SAXS tensor tomogram, with a voxel size of 25 µm^3^, was first interpolated to a voxel size of 20 µm^3^ using linear interpolation. Each 3D matrix of the spherical harmonics coefficient *a*_l_^m^ and of the three components of **û** ^str^ was translated and rotated around all three axes to match the reference frame of the sliced sample. Subsequently, the direction of the vector **û** ^str^ in each voxel was rotated as well around all three axes using a rotation matrix equivalent to the aforementioned rotations. For the validation of the nano­structure orientation, the absolute value of the dot product between the unit vector from SAXS tensor tomography and 3D sSAXS was calculated in each voxel.
